# Effect of Dexmedetomidine Low Doses with or without Midazolam in Cats: Clinical, Hemodynamic, Blood Gas Analysis, and Echocardiographic Effects

**DOI:** 10.1155/2022/9613721

**Published:** 2022-11-24

**Authors:** Marina Lopes Castro, Bruna Maia Cerqueira Câmara, Maira Souza Oliveira Barreto, Raphael Rocha Wenceslau, Andressa Karollini e Silva, Natália Fagundes, Renata Andrade Silva, Eutálio Luiz Mariani Pimenta, Suzane Lilian Beier

**Affiliations:** ^1^Animal Science Post Graduation Program, Federal University of Minas Gerais, Departament of Veterinary Medicine and Surgery, Belo Horizon, Minas Gerais, Brazil; ^2^Veterinary Internal Medicine and Surgery Department, Federal University of Minas Gerais, Belo Horizon, Minas Gerais, Brazil

## Abstract

**Objectives:**

The aim of the study is to compare the sedative, cardiorespiratory, echocardiographic, and blood gas effects of dexmedetomidine and methadone associated or not with midazolam for restraint chemistry in cats.

**Methods:**

Eighteen healthy young cats (4.06 ± 0.48 kg) were randomly sedated with two protocols, through the intramuscular route: dexmedetomidine (5 *µ*g.kg^−1^), methadone (0.3 mg. kg^−1^) and midazolam (0.3 mg. kg^−1^) (DMTM, *n* = 9), or dexmedetomidine (7.5 *µ*g.kg^−1^) and methadone (0.3 mg. kg^−1^) (DMT, *n* = 9). The cardiorespiratory parameters were measured at baseline, 5 and 10 minutes after pharmacological latency. The sedation, analgesia, and muscle relaxation scores were assessed before and 5 minutes after pharmacological latency, while arterial blood gas analysis and echocardiography were assessed before and after 10 or 15 minutes, respectively.

**Results:**

There was no difference between the protocols regarding the cardiorespiratory, blood gas, and echocardiographic parameters used. The scores for sedation, analgesia, and muscle relaxation also did not differ between the protocols, with the degree of sedation, analgesia, and myorelaxation considered satisfactory in both groups. A significant decrease in heart rate (HR) was observed after administration of the sedative protocols, reaching a maximum reduction at T10 (46% and 53% reduction in the DMT and DMTM groups, respectively). The reduction in HR had an impact on echocardiographic parameters such as CO, which decreased 53% and 56% in the DMT and DMTM groups, respectively. There was a significant reduction in PaO_2_, SaO_2_, ejection fraction, and fractional shortening in both protocols. SpO_2_ decreased significantly after 5 minutes of sedation in the DMT group, but with a minimum mean SpO_2_ of 92% in T5. The respiratory rate decreased significantly at 5 and 10 minutes in the DMTM group, while PaCO_2_ increased in both groups, indicating respiratory depression caused by the drugs. *Conclusions and Relevance*. The study pointed out that both sedative protocols can be recommended for clinical sedation of young and healthy cats in the doses used. However, both protocols resulted in cardiorespiratory depression in cats and also the particularities of the animals should be evaluated regarding reducing cardiac output by more than 50%.

## 1. Introduction

The physiological and morphological particularities of felines differ from the canine species mainly because these animals are less tolerant to handling and physical restraint [1–4].

Sedation in cats is often required for diagnostic procedures such as echocardiographic examination. The aim of sedation is lower stress, fear, and anxiety and also provide relaxation and when necessary, analgesia. [5] Furthermore, morbidity and mortality during anesthesia in this species are considerably higher as compared to dogs [6].

The use of *α*2-adrenergic receptor agonists brings benefits to clinical anesthesiology, especially dexmedetomidine, due to its pharmacological characteristics and high specificity. Dexmedetomidine is the pharmacologically active D-isomer of medetomidine, which is a highly selective agonist of the *α*2-adrenoceptor. It is not a pure *α*2-adrenoceptor agonist, as it is also able to bind to noradrenergic imidazoline receptors [7].

The cardiovascular effects of *α*2-adrenergic receptor agonists are likely to be one of the greatest concerns. By activating *α*-adrenergic receptors, these drugs cause significant vasoconstriction, increasing systemic vascular resistance and inducing reflex bradycardia [8]. Even lower doses of dexmedetomidine alone (5 *μ*g.kg−1) were reported to cause a decrease in cardiac output (CO) [9].

Midazolam, belonging to the benzodiazepine class, acts by enhancing the activity of the neurotransmitter GABA, the main inhibitory neurotransmitter of the central nervous system (CNS). The inclusion of this drug in the sedation protocol increases the sedative effect and provides greater intensity of muscle relaxation, with minimal interference in the cardiovascular system, when administered to dogs and cats [10].

The use of opioids, in therapeutic doses, interferes minimally with the cardiorespiratory system, although a decrease in heart rate can be noticed in some animals due to its parasympathomimetic effect [11]. According to The American Association of Feline Practitioners (AAFP) in 2018 [12], opioids are favorable in cats when used as preanesthetic medication, they promote analgesia, potentiate sedation and consequently reduce handling stress.

The association of different drugs during a sedative procedure is called neuroleptanalgesia and its greater advantage is the possibility of using combinations of lower dosages and in that way having less side effects and a safer protocol [13].

The study and development of safe protocols, with quick duration and with the possibility of reversion, is necessary within this specie, whose management involves significant stress and the need for restraint.

## 2. Objectives

The aim of the study was to compare the clinical effects such as heart rate, respiratory rate, blood pressure, pulse oximetry, and temperature. The echocardiographic changes, blood gas effects, and the quality of sedation were also compared. The protocols of sedation were dexmedetomidine and methadone associated or not with midazolam. These parameters indicate whether the protocols are safe and effective in causing effective chemical restraint in the feline.

## 3. Materials and Methods

### 3.1. Animals

Eighteen animals of the cat's species, healthy, male, Brazilian shorthair, aged between one to five years, with an average weight of 4.06 ± 0.48 kg. The animals evaluated were considered healthy and selected for the project after presenting results within normal values for the feline species in the clinical, laboratory (blood count, serum biochemistry, renal function), and echocardiographic (performed one week before the study, in case of a normal exam the measurements were considered as baseline). Animals were randomly divided into two groups, each one containing nine cats. The research project was approved by the Ethics Committee in the Use of Animals (CEUA) of the Federal University of Minas Gerais (Protocol no: 383/2017) and written informed consent was obtained from all owners upon admission of study cats to the hospital.

### 3.2. Animals Preparation

The experiment phase was carried out at the Veterinary Hospital of the Federal University of Minas Gerais. Food fasting of 12 hours and water fasting of 2 hours on the day of preparation and sedation was recommended.

General anesthesia was induced with isoflurane diluted in 100% oxygen, with the aid of a mask coupled to the circuit without partial gas reintroduction. The anesthetic plan was kept superficial, through the observation of clinical parameters, such as maintenance of protective reflexes and cardiorespiratory stability. A 22 G [1] catheter was aseptically introduced into the metatarsal artery to measure invasive blood pressure and to collect 0.5 mL of blood in a syringe with sodium heparin for blood gas analysis (pH, HCO3- (std), base deficit BE(ECF), PaCO2, SaO2, PaO2, sodium, potassium, chlorine, glucose, Hb, Ht, and lactate). The arterial catheter was filled with heparinized saline solution (0.9% NaCl with 5UI/mL of sodium heparin) to prevent it from clotting until the experimental protocol was carried out. After implantation of the arterial catheter, the supply of inhaled anesthetic was interrupted, and the animals were housed in individual metal cages 90 × 90 × 40 cm, in an airy room, with an average temperature of 25°C, until they recovered from the inhalation anesthesia. A minimum time of 60 minutes was followed between the animals' recovery and the administration of the drugs to be tested.

After administration of the sedation protocol and latency (the animal showed evident clinical sedation), the animal was positioned on the echocardiography table in the left lateral recumbence and disposable electrode adhesives for ECG were glued to the pads of both thoracic and left pelvic limbs for evaluating the electrocardiographic tracing and obtaining the heart rate. The arterial catheter was connected to a pressure transducer [2] positioned at the level of the heart to obtain systolic, mean, and diastolic blood pressure (SBP, MAP, and DBP). The pulse oximeter sensor was placed on the digit of one of the pelvic limbs to monitor peripheral oxygen saturation (SpO2), all connected to the multiparameter monitor [3]. The respiratory rate, in mov/min, was obtained by observing the movement of the rib cage per minute. Rectal temperature was measured using a digital clinical thermometer.

After shaving the left and right parasternal region, the echocardiographic evaluations were performed by an experienced examiner using the equipment with an electronic sectorial scan transducer (8.0–3.0 MHz) and with electrocardiographic monitoring on the equipment monitor [4]. The echocardiographic examination was performed using two-dimensional mode, *M* mode, pulsed Doppler (PW), continuous (CW), color flow mapping (CFM), and tissue Doppler, as recommended by the Echocardiography Committee of the Specialty of Cardiology-American College of Veterinary Internal Medicine [14] with modifications suggested by Boon J. A [15].

In the bidimensional mode measured, the diameter of the aorta (Ao) in the right parasternal cross section at the level of the heart base [16]. In the same section (M mode), at the level of the tendinous strands, the internal diameters of the left ventricle end-diastolic (LVIDd) and end-systolic (LIVDs) were measured and used to calculate the fractional shortening (FS%) using the formula FS% = [(LVIDd - LIVDs)/LVIDd]X 100 (LOMBARD, 1984) and the ejection fraction in *M* mode (FE%). The end-systolic and diastolic volumes (VSF and VDF, in mL) were calculated, using the Teichholz method, using the formulas VSF = [7/(2.4+LVIDs)](LVIDs [3]) and VDF = [7/(2, 4+LVIDd)](LVIDd [3]), with the LVIDd and LVIDs in cm. Afterward, the stroke volume (SV, in mL) and cardiac output (CO, in L/min) were calculated using the formulas SV = EDV-ESV, and CO = SV *x* FC. Ejection fraction (EF) was also evaluated by the Simpson method, interventricular septum thickness in diastole (IVSD) and in systole (IVSS), the posterior wall of the left ventricle in diastole (LVPWD) and in systole (LVPWS)).

In the four-chamber apical view, the transmitral and trans tricuspid flow were acquired and the maximum velocity peaks of the E wave (early ventricular filling), and of the A wave (late ventricular filling) were measured and the E/A wave ratio was calculated. The isovolumic relaxation time (IVRT) was obtained in the apical five-chamber view through an intermediate flow between the mitral inflow and the aortic flow [17]. Pulsed tissue Doppler was used to acquiring velocity waves derived from myocardial motion, with Em (rapid ventricular filling) and Am (late ventricular filling) obtained by apical four-chamber view, with the sample volume positioned on the free wall of the left ventricle in the mitral (parietal) annulus. The relationships between transmitral flow E wave and peak tissue velocity parietal Em (E/Em) and parietal Em/Am were calculated [17]. The peak pulmonary artery flow (P) and the right (TD-r) and left (TD-l) tissue Doppler were also evaluated.

Assessments of the quality of sedation, muscle relaxation, and posture were performed by an evaluator, who was unaware of the treatment used, assigning scores for the quality of sedation, degree of muscle relaxation, and posture scores, as described in Table 1.

Latency time, in minutes, was established as the time from drug administration until visible sedation was observed or 10 minutes after drug administration. The duration of sedation, in minutes, was established as from the beginning of latency to the beginning of spontaneous movement of the animals (considering the lifting of the head and movement of the limbs). And the time for full recovery, in minutes, was defined as the time between the end of sedation (considering lifting the head and moving the limbs) until the spontaneous movement of the animals and ambulation occurred.

### 3.3. Experimental Protocol

After acclimatization at the site of the echocardiographic evaluation, the baseline variables (BL) were collected: cardiorespiratory parameters (HR, *f*, SBP, MAP, DBP, SpO2), T^o^C, arterial blood gas analysis and sedation, muscle relaxation and nociception scores. Then, the following sedation protocols were administered, randomly by drawing, by the intramuscular route in a single syringe: dexmedetomidine [5] (5 *µ*g.kg−1), methadone [6] (0.3 mg.kg−1), midazolam [7] (0.3 kg−1) (DMTM, *n* = 9), or dexmedetomidine (7.5ug.kg−1) and methadone [6] (0.3 mg.kg−1) (DMT, *n* = 9).

Five minutes after drug latency (time between administration and presentation of evident clinical sedation or 10 minutes after drug administration), cardiorespiratory parameters and T^o^C (T5) were evaluated again by the same evaluator, and every 5 minutes until completed 10 minutes after latency (T5 and T10, respectively). Sedation, muscle relaxation, and nociception scores were performed again 5 minutes after drug latency time. Arterial blood gas analysis was repeated at T10 (10 minutes after latency) and echocardiographic evaluation was performed at T15 (15 minutes after latency).

### 3.4. Statistical Analysis

To evaluate the effect of anesthetic protocol, time, and the interaction between these factors on the variables studied, a mixed model was adjusted considering the protocol and time as fixed effects and animals as a random effects in order to account for repeated measurements of the same individual. The means of the groups were compared using the Tukey test. To evaluate the variables that did not assume the assumptions of the analysis of variance, such as sedation, analgesia, and muscle relaxation scores, as well as recovery time, the Wilcoxon test was used to compare the times and the Mann–Whitney test to compare groups. The analyses were performed using the *R* 3.6.1 software (R Core Team, 2020). A significant difference was considered when *p* ≤ 0.05. To describe the association between methods for obtaining stroke volumes (systolic volume) and cardiac output, Spearman's correlation was used using the Graph Pad Prism® computer program for Mac OSX Version 8.2.1.

## 4. Results

There was no difference between the protocols regarding the latency time and the animals' sedation time. The total recovery time was longer in the animals in the group that received midazolam (DMTM) compared to the DMT group. The mean and standard deviation of latency, sedation, and recovery times (in minutes) are shown in Table 2.

**Figure 1:**
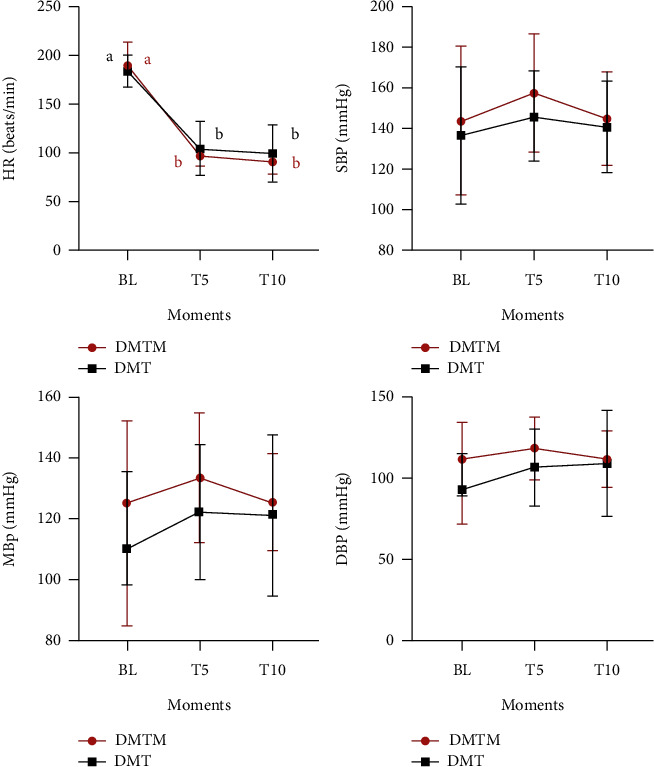
Values of heart rate (HR) and systolic, diastolic, and mean blood pressure (SBP, DBP, MBP, respectively), in 18 felines sedated with the association of dexmedetomidine (7.5 ug/kg) and methadone (0.3 mg/kg) (DMT, *n* = 9) or dexmedetomidine (5 ug/kg), methadone (0.3 mg/kg), and midazolam (0.3 mg/kg) (DMTM, *n* = 9), both by the intramuscular route. Values are presented as mean ± standard deviation. a, b: different lowercase letters indicate significant differences between moments inside the same group according to Tukey test (*p* < 0.05).

**Figure 2:**
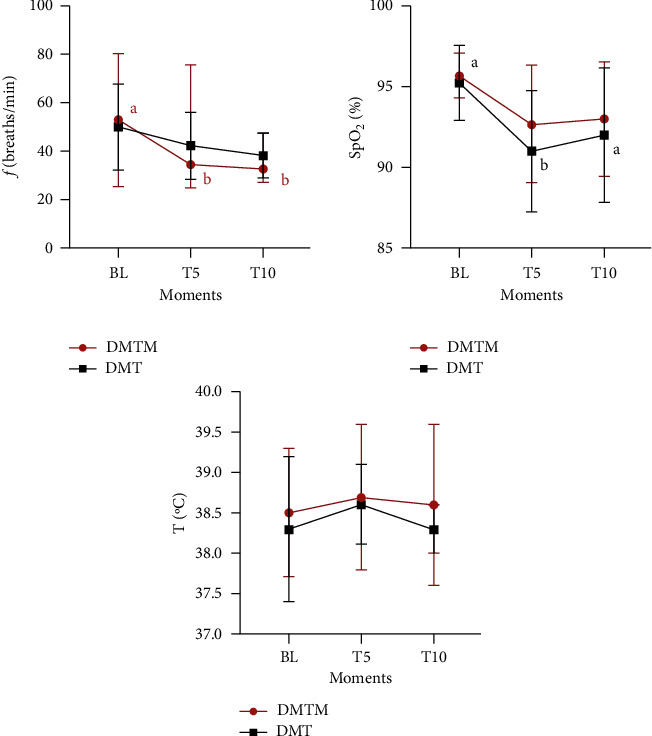
Values of respiratory rate (f), hemoglobin peripheral saturation thorough oxygen (SpO_2_), and rectal temperature (T°C), in 18 felines sedated with the association of dexmedetomidine (7.5ug/kg) and methadone (0.3 mg/kg) (DMT, *n* = 9) or dexmedetomidine (5ug/kg), methadone (0.3 mg/kg), and midazolam (0.3 mg/kg) (DMTM, *n* = 9), both by the intramuscular route. Values are presented as mean ± standard deviation. a, b: different lowercase letters indicate significant differences between moments inside the same group according to Tukey test (*p* < 0.05).

The clinical parameters evaluated (HR, SBP, DBP, MAP, SpO2, *f,* and T°C) did not differ between the studied protocols at any of the evaluated moments (BL, T5, and T10) (Figure 1; Figure 2). Heart rate decreased significantly at T5 and T10 when compared to baseline in both groups, with a maximum reduction of 46% and 53% at T10 for DMT and DMTM, respectively, but with no difference between T5 and T10 moments (Figure 1). There was no significant increase in systolic, diastolic, and mean blood pressure after the administration of sedative protocols in both groups (Figure 2). There was a reduction in peripheral hemoglobin oxygen saturation only in the DMT group at time T5 when compared to baseline. The respiratory rate decreased significantly in the DMTM group at T5 and T10 compared to baseline. There was no significant difference in the body temperature of the animals in both groups, in the evaluated moments (Figure 2).

There was no difference in the time, in minutes, to perform the echocardiogram between the two groups (14.31 ± 5.66 and 11.6 ± 2.85 minutes for the DMT and DMTM groups, respectively). There was no difference between the two protocols in any of the echocardiographic parameters evaluated.

**Figure 3:**
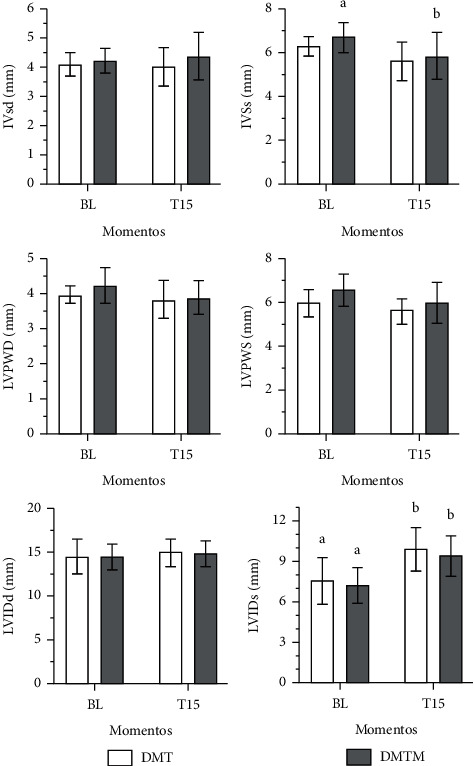
Values for interventricular septum in diastole (IVSd) and systole (IVSs), left ventricular posterior wall in diastole (LVPWD), and systole (LVPWS) and left ventricular internal diameter in diastole (LVIDd) and systole (LVIDs), in 18 sedated cats with the combination of dexmedetomidine (7.5ug/kg) and methadone (0.3 mg/kg) (DMT, *n* = 9) or dexmedetomidine (5ug/kg), methadone (0.3 mg/kg), and midazolam (0.3 mg/kg) (DMTM, *n* = 9), both by the intramuscular route. Values are presented as mean ± standard deviation. a, b: different lowercase letters indicate a significant difference between moments within the same group according to the Tukey test (*p* < 0.05).

**Figure 4:**
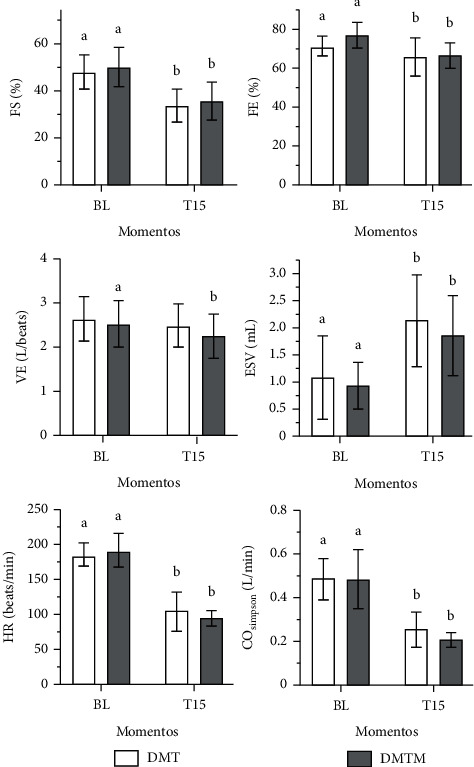
Values for shortening fraction (FS%), ejection fraction by Simpson (FE), heart rate (HR), end-systolic volume (ESV), ejection volume by Simpson (VE), and cardiac output by Simpson (CO), in 18 cats sedated with the association of dexmedetomidine (7.5 ug/kg) and methadone (0.3 mg/kg) (DMT, *n* = 9) or dexmedetomidine (5 ug/kg), methadone (0 .3 mg/kg), and midazolam (0.3 mg/KG) (DMTM, *n* = 9), both by the intramuscular route. Values are presented as mean ± standard deviation. a, b: Different lowercase letters indicate a significant difference between moments within the same group according to the Tukey test (*p* < 0.05).

**Figure 5:**
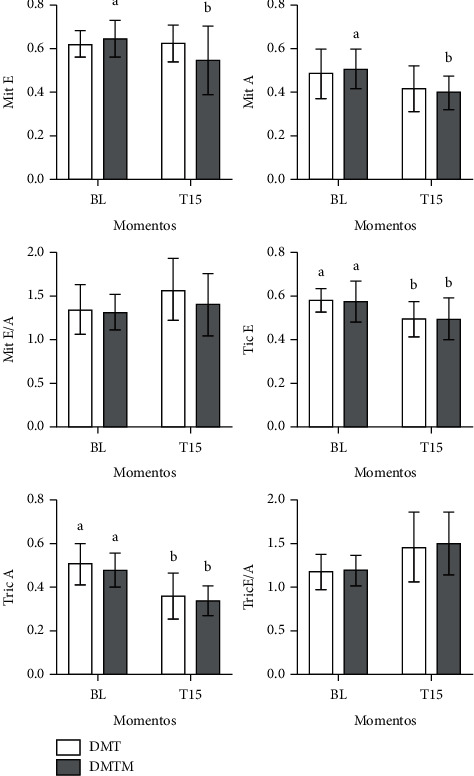
Values of E wave (E) and A wave (A) in the mitral valve, ratio of the E/A wave in the mitral (Mit E/A), E wave (tric (E)), and A wave (tricus (A)) in the tricuspid valve, and tricuspid E/A wave ratio (tric E/A) in 18 cats sedated with the combination of dexmedetomidine (7.5 ug/kg) and methadone (0.3 mg/kg) (DMT, *n* = 9) or dexmedetomidine (5 ug/kg), methadone (0.3 mg/kg), and midazolam (0.3 mg/kg) (DMTM, *n* = 9), both by intramuscular route. Values are presented as mean ± standard deviation. a, b: different lowercase letters indicate a significant difference between moments within the same group according to the Tukey test (*p* < 0.05).

**Figure 6:**
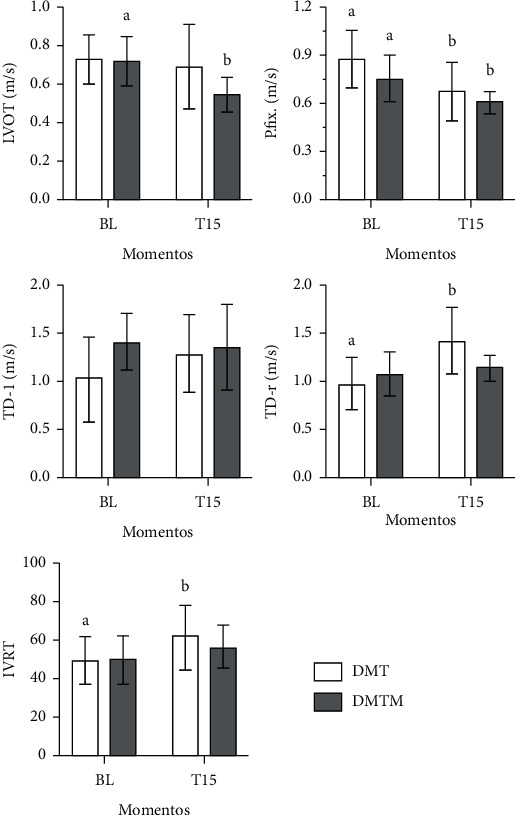
Values of left ventricular outflow tract (LVOT) velocity, pulmonary artery flow (P. flx), isovolumic relaxation time (IVRT), left tissue Doppler (TD-l), and right tissue Doppler (TD-r), in 18 felines sedated with the combination of dexmedetomidine (7.5 ug/kg) and methadone (0.3 mg/kg) (DMT, *n* = 9) or dexmedetomidine (5 ug/kg), methadone (0.3 mg/kg), and midazolam (0.3 mg/kg) (DMTM, *n* = 9), both by the intramuscular route. Values are presented as mean ± standard deviation. a, b: different lowercase letters indicate a significant difference between moments within the same group according to the Tukey test (*p* < 0.05).

Heart rate at T15 was lower compared to baseline values (56% and 51% reduction in the DMT and DMTM groups, respectively). There was a significant reduction in Simpson-mode ejection fraction (8.5% and 14%, in the DMT and DMTM group, respectively), as well as in the M-mode ejection fraction (21% and 18%, in the DMT and DMTM group, respectively), in both groups, 15 minutes after the pharmacological latency of the sedation protocols. The measurement of the interventricular septum in systole (IVS) decreased only in the DMTM group. The measurement of left ventricular internal diameter at systole (LVIDs) increased significantly, on average 31%, in both groups. The shortening fraction reduced, on average, 30% and 28% in the DMT and DMTM groups, respectively. There was a 44% and 51% reduction in heart rate values in the DMT and DMTM groups after 15 minutes of pharmacological latency, compared to baseline values. The end-systolic volume (ESV) value increased significantly at T15 in both groups. Stroke volume differed in the TMD group, showing a reduction in this parameter at time T15 compared to baseline. Pulmonary artery flow velocity (P) decreased significantly at time T15 in both groups. A 27% increase in IVRT was observed only in the DMT group after 15 minutes of pharmacological latency. In the right tissue Doppler there was a significant increase of 45% in the DMT group after 15 minutes of sedation. There was a decrease in E (Mit E) and A (Mit A) waves of the mitral valve in the DMTM group. The other echocardiographic parameters, such as IVSd, LVIDd, LVPWD, LVPWS, EDV, Ao, Mit E/A, Tric E/A, and TD-l showed no difference either in comparison between treatments or in relation to the baseline value (Figures 3–6).

**Figure 7:**
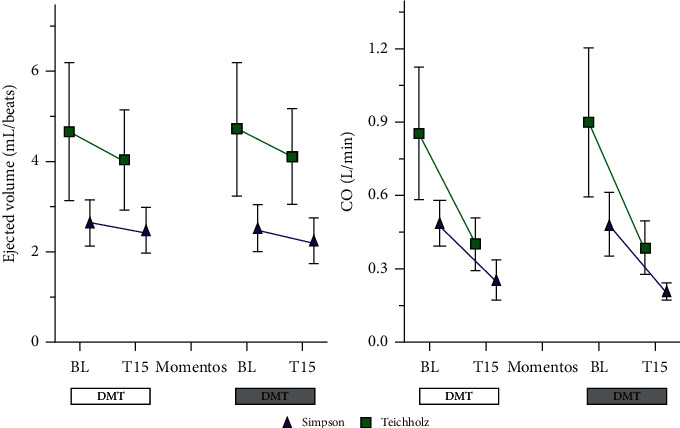
Values of stroke volume (ejected volume) and cardiac output (CO), obtained by the Simpson and Teichholz methods, in 18 cats sedated with the association of dexmedetomidine (7.5 ug/kg) and methadone (0.3 mg/kg) (DMT, *n* = 9) or dexmedetomidine (5ug/kg), methadone (0.3 mg/kg), and midazolam (0.3 mg/kg) (DMTM, *n* = 9), both by the intramuscular route. Values are presented as mean ± standard deviation.

**Figure 8:**
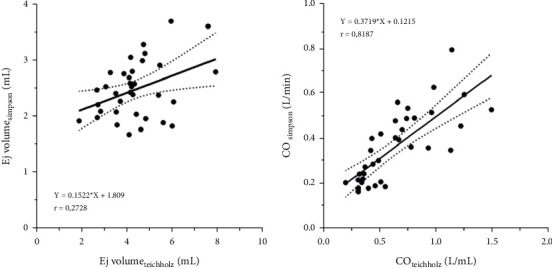
(a) Spearman correlation between the ejected volume obtained by the Teichholz and Simpson methods. The correlation obtained had *r* = 0.27 of 18 measurements. (b) Spearman correlation between cardiac output (CO) obtained by the methods of Teichholz and Simpson. The correlation obtained had *r* = 0.82 out of 18 measurements.

The comparison between the values obtained for stroke volume and cardiac output using the Teichholz and Simpson methods is shown in Figure 7. Both the stroke volume and the cardiac output were higher by the Teichholz method compared to the Simpson method (that is, a tendency to overestimate the data by Teichholz when compared to Simpson). Through the analysis of the Spearman correlation test, there was a weak association between the ejected volume measurements in both the methods (*r* = 0.27), however, the CO values showed a strong association (*r* = 0.82) (Figure 8).

**Figure 9:**
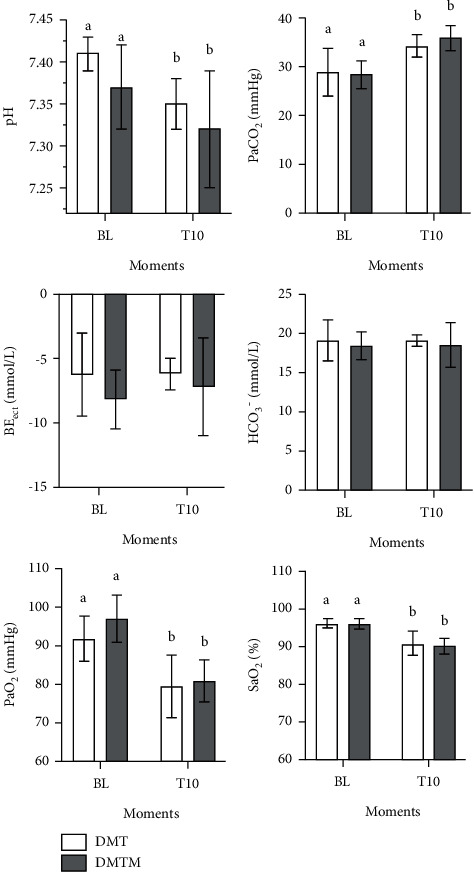
Values of arterial pH, arterial carbon dioxide, and oxygen tensions (PaCO_2_ and PaO_2_, respectively), arterial oxygen saturation (SaO_2_), excess/deficit of bases (BE), and bicarbonate (HCO3−), in 18 cats sedated with the association dexmedetomidine (7.5 ug/kg) and methadone (0.3 mg/kg) (DMT, *n* = 9) or dexmedetomidine (5 ug/kg), methadone (0.3 mg/kg), and midazolam (0.3 mg/kg) (DMTM, *n* = 9), both by the intramuscular route. Values are presented as mean ± standard deviation. a, b: different lowercase letters indicate a significant difference between moments within the same group according to the Tukey test (*p* < 0.05).

**Figure 10:**
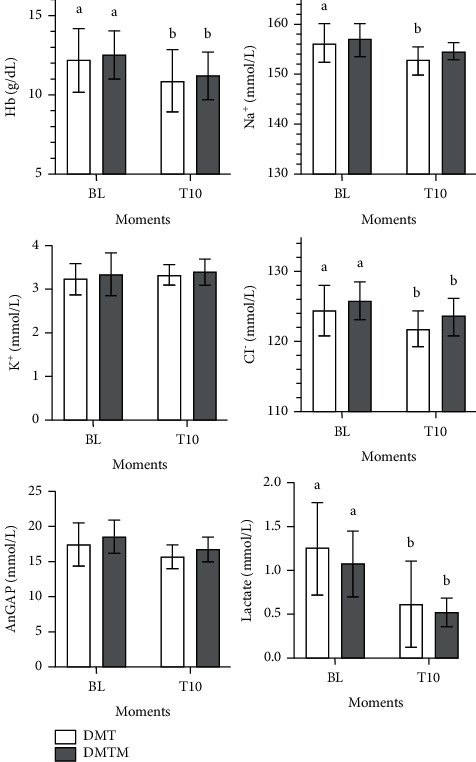
Values of arterial hemoglobin (Hb), arterial plasmatic sodium, potassium, and chloride (Na+, *K*+, Cl−, respectively), anion gap concentration (AnGAP), and arterial lactate plasma concentration (lactate) in 18 felines sedated with the association of dexmedetomidine (7.5 ug/kg) and methadone (0.3 mg/kg) (DMT, *n* = 9) or dexmedetomidine (5 ug/kg), methadone (0.3 mg/kg), and midazolam (0.3 mg/kg) (DMTM, *n* = 9), both by the intramuscular route. Values are presented as mean ± standard deviation. a, b: different lowercase letters indicate a significant difference between moments within the same group according to the Tukey test (*p* < 0.05).

Blood gas parameters did not differ between protocols (Figure 9). There was a decrease in pH at T10 compared to baseline in both groups. Arterial carbon dioxide pressure (PaCO2) increased after 10 minutes of drug latency in both protocols. There was a reduction in arterial oxygen pressure (PaO2), arterial oxygen saturation (SaO2), and hemoglobin (Hb) in both groups at T10. Arterial sodium concentration (Na+) was reduced only in the DMT group. Arterial chlorine (Cl−) concentration decreased in both groups, with a significant difference compared to baseline. There was a reduction in lactate when compared to baseline values in both protocols at the time evaluated (T10). There was no difference in the parameter's bicarbonate (HCO3−), excess of bases (BE), potassium concentration (*K*+), and anion gap (Figure 10).

**Figure 11:**
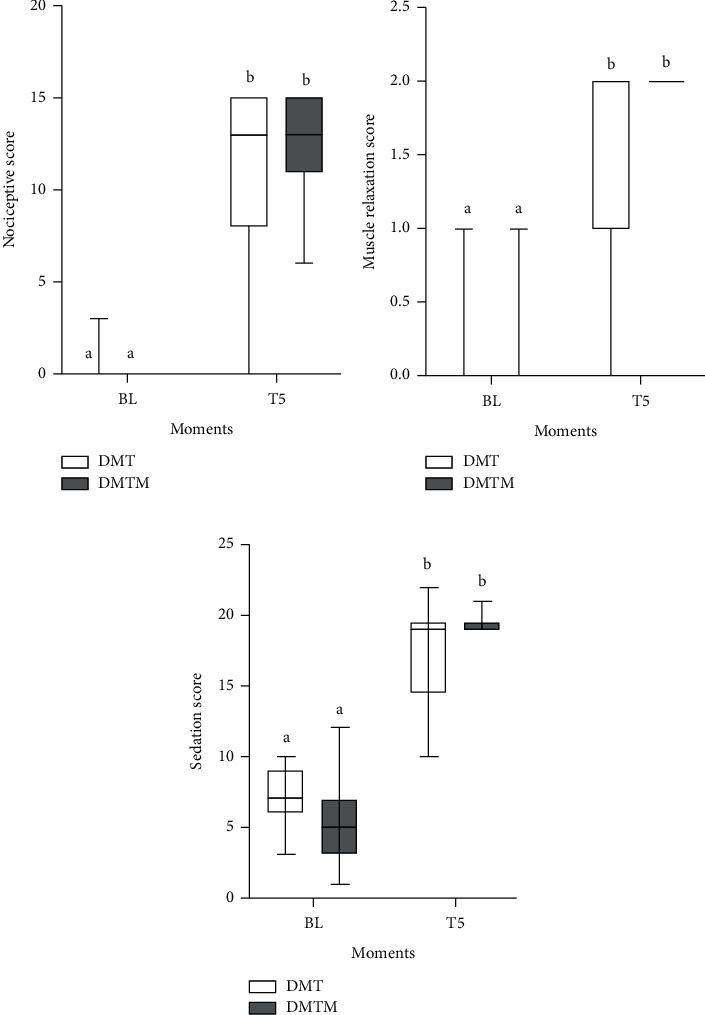
Sedation scores, nociceptive response, and muscle relaxation were observed in 18 cats sedated with the combination of dexmedetomidine (7.5 ug/kg) and methadone (0.3 mg/kg) (DMT, *n* = 9) or dexmedetomidine (5 ug/kg). kg), methadone (0.3 mg/kg), and midazolam (0.3 mg/kg) (DMTM, *n* = 9), both by the intramuscular route. Maximum scores for possible sedation, nociceptive response, and muscle relaxation are 19, 15, and 2, respectively. Values are presented as “boxplots,” with 50% of the data contained in the box. The bold horizontal line represents the median and the bars represent the score variation. a, b: different lowercase letters indicate a significant difference between moments within the same group according to the Wilcoxon test (*p* < 0.05).

Sedation, nociceptive response, and muscle relaxation scores did not differ between protocols. After 5 minutes of drug latency, both groups obtained sedation, nociception, and myorelaxation scores compatible with deep sedation (Figure 11).

## 5. Discussion

Our main goal was to establish a sedation protocol for felines that promotes adequate chemical restraint with minimal alterations in echocardiographic, blood gas, and clinical parameters.

In this study, sedation was effective for both protocols, but with important changes in cardiorespiratory and echocardiographic parameters, such as bradycardia, moderate hypoxemia, reduced cardiac output, and systolic function. In addition, the results of this research suggest that there were no differences in the thickness of the diastolic diameter of the ventricular free wall and the interventricular septum, thus not affecting the diagnosis of hypertrophic cardiomyopathy in felines.

Comparing the mean baseline parameters, such as HR, *f*, SBP, DBP, and MBP, with other studies that evaluated these same baseline parameters in felines [18–20], no discrepancies were observed between them, suggesting that the animals were not overly stressed or agitated.

The total recovery period was longer in the animals in the DMTM group that received midazolam compared to the DMT group. The difference observed for the recovery period was also observed in the article by Kanda and Hikasa [21] using the association of medetomidine and midazolam. The increase in recovery time can be explained by the fact that midazolam increased the plasma half-life.

The reduction in HR in both groups can be explained by dexmedetomidine associated with methadone. Regarding the bradycardia caused by alpha 2 agonists, these drugs activate presynaptic receptors in peripheral nerve endings, with a reduction in the release of norepinephrine and the sympatholytic effect on the CNS [22]. In the dexmedetomidine action phase, baroreflex activity occurs with a decrease in sympathetic tone and consequent decrease in heart rate, corroborating the data found in this study in relation to the decrease in HR [23, 24]. The association of methadone to the protocols, seen in this project, may have contributed even more to the drop-in heart rate since opioids have parasympathomimetic effects [11]. According to Kukanich and Wiese [25], the cardiovascular effects of opioids are potentiated when they are administered concomitantly with drugs that affect cardiac output and vascular resistance, such as *α*2-adrenergic receptor agonists. If we compare the DMT and DMTM protocols, we can infer that the depressant effects on the cardiovascular system do not appear to be dose-dependent.

Several reports using dexmedetomidine alone or in combination with other drugs showed divergent results regarding the effect on blood pressure in cats [13, 26, 27]. These discrepancies in the results of the studies can be explained by the use of different doses, the measurement time of this parameter, as well as the drug combinations that differ between the articles. In addition, the different methods of measuring blood pressure and stress levels in cats across studies may also affect the results [19, 28]. In the present project, a 50% higher dose of dexmedetomidine in the DTM group did not result in higher SBP, DBP, and MBP values compared to DMTM, suggesting that hypertension, at the doses used, does not occur in a dose-dependent manner.

Low values of SpO2, *f*, PaO2, and SaO2 and high values of PaCO2 were observed indicating moderate hypoxemia and respiratory depression [29]. Leppänen [30] reported that higher doses of dexmedetomidine, as well as other *α*2-adrenergic agonists, cause a dark red or pale color on the tongue, due to peripheral vasoconstriction, which consequently can cause difficulty in reading the oximeter due to the reduction in peripheral perfusion. Thus, positioning the pulse oximeter on the tongue in animals sedated with these drugs will result in borderline values or severe hypoxemia. In this project, the oximeter was positioned on the digit, which could also cause reading difficulties due to peripheral vasoconstriction. Another factor that the research by Leppänen [30] cited that may explain the drop-in oximetry in patients is respiratory depression caused by sedation with the use of alpha-2-agonist.

The occurrence of respiratory depression is mainly observed by the reduction of respiratory movements, resulting in an increase in PaCO2, induced by the action of the drug in the upper respiratory centers due to the distribution of *α*2 adrenoreceptors in the brain [31, 32]. In addition, Flôres et al [33] suggest that midazolam may reduce the ventilatory response, leading to increased PaCO2 and central respiratory depression. Therefore, this probable synergistic or additive depressant effect on the respiratory center could explain the significant reduction in the respiratory rate in animals that received midazolam (DMTM).

There was no significant change in rectal temperature in any of the protocols used over the time evaluated. In the article by Selmi et al. [13], the rectal temperature of cats decreased significantly compared to baseline after 40 minutes of sedation. The short evaluation time of the research project may have been a limitation for not having observed changes in the animal's rectal temperature.

Regarding sedation, nociceptive response, and muscle relaxation scores according to the simple descriptive analysis table (adapted from Ansah et al., [34]), there was no difference between the protocols. Both protocols achieved a satisfactory degree of sedation, muscle relaxation, and analgesia. According to the article by Selmi et al. [13], the combination of an *α*2-agonist with an opioid in cats resulted in a greater degree of sedation compared to the use of an *α*2-agonist alone.

Ansah et al., [34] concluded that the use of dexmedetomidine in cats at doses (25, 50, and 75 *μ*g/kg) induces, in a dose-dependent manner, sedation, analgesia, and clinically important muscle relaxation. An article comparing the use of dexmedetomidine alone or in combination with various opioids in healthy dogs reported that the sedative effects of dexmedetomidine were more pronounced when combined with a variety of opioids, particularly with butorphanol, meperidine, and methadone, compared with the sedative effects when dexmedetomidine was administered alone [35]. These studies justify the excellent degree of sedation that the present research obtained in both protocols, noting that the association of dexmedetomidine with opioids, such as methadone, associated or not with midazolam potentiates the sedative degree, allowing the use of low doses of drugs.

According to our research, the group that received midazolam did not obtain more intense muscle relaxation scores than the other group, as predicted in the literature. This fact may have occurred due to a limitation of the muscle relaxation scale used, which ranges from 0 (not relaxed) to 2 (very well relaxed), that is, 3 possible classifications. However, if we to analyze the individual values, 100% of the DMTM group had a maximum score, compared to 67% of the DMT group, indicating a probable better muscle relaxation when using midazolam associated with dexmedetomidine and methadone.

In the blood gas analysis, there was no difference between the groups. A significant drop was observed in both groups, compared to baseline values, for pH, PaO2, SaO2, Hb, Cl, and lactate. The respiratory depression caused by the drugs used in both protocols explains the increase in PaCO2, and consequently the fall in pH, due to the increase in carbonic acid caused by respiratory acidosis [36].

In the present study, lactate values remained within the normal range for the species, 0.5 to 2.0 mmol/L [37], both at BL and T10 times, in both groups. Hyperlactatemia occurs when lactate production exceeds metabolism and its elimination, especially in situations of activation of the hypothalamic-pituitary-adrenal axis with consequent vasoconstriction and tissue hypoxia. However, there was a significant reduction in lactate after 10 minutes of pharmacological latency (T10) compared to baseline in both groups. Biermann et al. [38] justified this observation by the fact that sedation provides muscle relaxation and stress reduction, decreasing lactate concentrations.

There was a decrease in Hb concentration after sedation with both protocols. The reduction of red blood cells and total hemoglobin concentration was also observed in the study by Biermann et al. [38]. In conscious animals, catecholamines respond to stress through splenic contraction leading to an increase in hemoglobin. This spleen response is minimized with the use of sedative drugs or associations with properties that reduce sympathetic activity, such as alpha-2-agonists, inducing a reduction in Hb values [39]. In contrast, the report by Congdon et al. [40], which evaluated the use of dexmedetomidine (10*μ*/kg) alone in dogs, did not observe a reduction in hemoglobin concentration after sedation with this drug.

Na + decreases significantly in the DMT group at time T5. Cl-reduced significantly in both groups but remained within the normal range for the species. Reductions in these electrolytes have not been observed with the use of dexmedetomidine alone in dogs [40]. As in the publication by Volpato et al. [41], who also did not observe changes in chloride after sedation of felines, using the protocols dexmedetomidine (5 *μ*g/kg) + butorphanol (0.3 mg/kg) and dexmedetomidine (5 *μ*g/kg) + butorphanol (0.3 mg/kg) + ketamine (3 mg/kg). There was no significant difference in the other electrolyte values obtained through blood gas analysis in this study.

In the present study, the animals were selected after verifying that they did not have any heart disease. In addition, they also did not show signs of thromboembolism, such as left atrial dilatation (LA longitudinal diameter<1.5 cm) and the presence of spontaneous contrast within the atrial chambers.

Postsedation echocardiographic variables with DMT and DMTM were compared with presedation values and between protocols, in healthy cats. There was an increase in ventricular diameters, and a reduction in stroke volume and cardiac output. The other echocardiographic parameters, such as IVDd, LIVDd, LVPWd, LVPWs, EDV, Ao. Mit E/A, tric E/A, and TD-l showed no difference either in comparison between treatments or in relation to the baseline.

In this research, both the ejection fraction and the shortening fraction reduced in relation to baseline, with significance, but there was no difference between the protocols. The FS% and EF% are indexes that quantitatively asses the left ventricular systolic function and demonstrate myocardial contractility, which is easily altered due to changes in HR, pre and postload, and contractility [15, 42]. Among these variables, HR has the least influence on systolic function.

The FS% is a percentage change in the dimension of the left ventricular cavity that occurs in systole [15]. In most cats, the FS% is 35 to 65%, although there is variability [43]. A low FS% value may be secondary to a decrease in preload, an increase in afterload, or a decrease in contractility. At work, there was no change in preload because the end-diastolic volume did not change, the increase in afterload may have influenced more significantly low values of FS% and a decrease in cardiac output.

The end-systolic volume is determined by cardiac contractility, by the blood volume that remains in the LV and the stroke volume corresponds to the amount of blood that leaves the heart, constituting a more accurate way to asses myocardial contractility in the presence of mitral regurgitation [43]. In this study, there was a limitation in the calculation of systolic and end-diastolic volumes using the Simpson method due to poor contact between the device and the HR electrodes, making the value and calculation by this method unavailable to the software.

The *α*2 agonists promote a sympatholytic effect, with depression of the vasomotor center, an increase in vagal tone and baroreceptor activity [44], thus, a reduction in myocardial contractility is observed. In the present study, there was an increase in end-systolic volume (ESV) in both groups, which may suggest that the dose and the drug combinations used in this study negatively interfered with myocardial contractility and did not interfere with the increase in afterload, as in our work the PAS did not change. In the article by Biermann et al. [45], a similar increase in FSV was observed after sedation of cats in protocols in which dexmedetomidine (5 *μ*g/kg) was inserted. The authors also justified this result in view of the particularities of alpha-2-adrenergic agonists in reducing heart contractility or increasing afterload through the vasoconstriction that these drugs cause. The increase in the end-systolic volume results in a decrease in the stroke volume, which together with the decrease in heart rate caused by alpha-2-agonists, corroborates the decrease in cardiac output [23].

Cardiac output is the result of heart rate multiplied by stroke volume (stroke volume), Kitahara et al. [46] reported a reduction in bradycardia-related CO and CI and an increase in peripheral vascular resistance attributed to the direct vasoconstriction effects of *α*2-adrenergic agonists. In the study by Biermann et al. (2012) [45], the combinations of dexmedetomidine (5 *μ*g/kg) + midazolam (0.4 mg/kg) + butorphanol (0.4 mg/kg) and dexmedetomidine (5 *μ*g/kg) + ketamine (3 mg/kg), reduced cardiac output by 54% and 53%, respectively. Corroborating these results, the current research reveals a significant reduction in this variable in both groups at T15, with no difference between the protocols. In the DMT group, the CO was reduced by 53%, while in the DMTM group by 56% in relation to baseline measurements, with no difference between the groups.

A human study that evaluated the agreement between LV stroke volume measurements, obtained by three-dimensional color mapping, three-dimensional volumetric variation, Teichholz's formula, modified Simpson's method, and Doppler estimation, observed that there was a significant linear correlation between the measurements of stroke volume by three-dimensional color mapping and measured by Doppler (*r* = 0.83), Simpson's rule (*r* = 0.87) and three-dimensional volumetric variation (*r* = 0.93), with *P* < 0.01 for all. On the other hand, there was no significant correlation with the Teichholz method (*r* = 0.30, *P*=0.3) [47]. In the present study, the correlation of the volume ejected by the Teichholz and Simpson methods was also low (*r* = 0.27). However, the CO obtained by the two methods showed a strong correlation (*r* = 0.82). Biermann, et al.,) [45] evaluated the agreement and repeatability of four echocardiography methods to measure stroke volume and cardiac output in cats and observed that the Teichholz and Trace (flow) methods were acceptable and repeatable, suggesting that these provide more representative values, so they could be the most useful methods for measuring stroke volume and cardiac output in cats.

Stroke volume was not different between the protocols, however, in the DMTM group, stroke volume by the Simpson method was significantly reduced, which did not happen with the TeichHolz method. However, the percentage reduction in stroke volume in the DMTM group was similar between the methods, being 12% and 11.4% in the Teichholz and Simpson methods, respectively. The fact that the mean and standard deviation were lower in the Simpson method both at baseline (2.54 ± 0.51) and at T15 (2.25 ± 0.50), compared to Teichholz (4.73 ± 1 .48) and (4.12 ± 1.05), baseline and T15, respectively, may have contributed to a significant reduction in stroke volume in the DMTM group, although clinically the percentage reduction between the methods was similar. As the stroke volume reduction observed was not intense, this may suggest that the CO reduction was mainly influenced by contractility and by the reduction in heart rate.

LV diastolic function comprises the chamber's ability to accommodate adequate blood volume to maintain cardiac output and meet metabolic demand. Tissue Doppler is an echocardiographic method associated with the assessment of left and right ventricular diastolic function to obtain the relationship between the E′ and A' (E'/A′) wave velocities that are measured both in the left ventricular free wall, at the level of the mitral valve insertion, and in the right ventricular free wall, at the level of the tricuspid valve insertion. This relationship makes it possible to demonstrate changes in the speed of movement of the heart muscle [48, 49]. Variations of these indexes beyond the limit of normality point to diastolic dysfunction and, depending on the pattern of abnormality, it can be inferred that the individual has impaired ventricular relaxation, that is, the active phase of diastole, or that he or she has less ventricular compliance/distensibility, in other words, of the passive phase of diastole [50]. Tissue Doppler ratio values (TD-l and TD-r) when <1 indicate diastolic dysfunction, which in this research did not happen after the administration of the protocols, therefore, there was no impairment of diastolic function with the drugs and doses that were used in both protocols.

By positioning the sample volume between the septal leaflet of the mitral valve and the left ventricular outflow tract, the transmitral and aortic flows are simultaneously recorded, which allows obtaining the isovolumic relaxation time (IRVT) [15, 51]. In the present study, the significant increase of 27% in IVRT may have been influenced by the reduction in heart rate after sedation. Faster myocardial relaxation results in lower IVRT values, and prolonged relaxation leads to an increase in this parameter (Schober and Todd) [17]. According to Nishimura et al. [52], the higher the heart rate, the lower the IVRT value, as tachycardia and sympathetic stimulation can exacerbate isovolumic relaxation, reducing diastolic filling time and accelerating early diastolic elastic recoil of the left ventricle, the opposite happens when the heart rate drops significantly. This index is also influenced by volume overload conditions, leading to an increase in the isovolumic relaxation time [53].

## 6. Conclusions

The protocols evaluated in this research were safe, well-tolerated by the animals, and demonstrated similar sedation scores and clinical, blood gas, and echocardiographic parameters. They provided appropriate levels of sedation, analgesia, and muscle relaxation but also induced cardiorespiratory depression with a significant reduction in the cardiac output. Considering healthy young cats, the protocols can be recommended. However, more studies are needed for disease animals, especially those with cardiomyopathies.

## Figures and Tables

**Table 1:** Simple descriptive analysis of sedation, muscle relaxation, and analgesia in felines.

Sedation evaluation	Escore
Posture and stability	0–6
Lateral recumbence, incapable of standing. Looks sleepy and not easily woken with little stimulation.	6
Lateral recumbence, incapable of standing. Looks awake or if sleepy can easily be woken up with small stimulation. Occasionally makes small movements, but it is not capable of keeping its head up.	5
Lateral recumbence or sternal, but occasionally stands up or after stimulation. Looks tired, and can walk, but does not want to. Stays ataxic or moderately ataxic.	4
Its capable of standing up or walking more than 5 meters with moderate or light ataxia.	3
Walks with slight or none ataxia is able to run.	2
Walks and runs almost normally, with no signs of ataxia, or slight signs of weakness.	1
Walks and runs normally.	0

Resistance to be put in lateral recumbence	0–3
No resistance	3
Little resistance	2
Moderate resistance	1
Strong resistance	0

Resistance to stretching the hind limbs without clamping	0–3
No resistance	3
Little resistance	2
Moderate resistance	1
Strong resistance	0

Resistance to stretching the knees	0–2
No resistance	2
Little resistance	1
Strong resistance	0

Reaction to clapping	0–3
No reaction	3
Little reaction	2
Moderate reaction	1
Strong reaction	0

Degree of mandibular relaxation/mandibular resistance/ability to open the mouth	0–2
No resistance	2
Possible, but hard	1
Strong resistance, impossible	0

Total sedation	0–19
Nociceptive answer	Escore
Interdigital clamping	0–5
Little intensity of clamping and there is already withdrawal response.	0
A lot of clamping intensity (clamp locking) and there is withdrawal response.	3
No withdrawal response even with high-intensity clamping (clamp lock).	5

Tail clamping	0–5
Little intensity of clamping and there is already withdrawal response.	0
A lot of clamping intensity (clamp locking) and there is withdrawal response.	3
No withdrawal response even with high-intensity clamping (clamp lock).	5

Skin clamping	0–5
Little intensity of clamping and there is already withdrawal response.	0
A lot of clamping intensity (clamp locking) and there is withdrawal response.	3
No withdrawal response even with high-intensity clamping (clamp lock).	5

Total of nociceptive evaluation:	0–15
Muscular relaxation	Escore
Very well relaxed.	2
Moderately well relaxed.	1
Not relaxed, normal	0

^*∗*^Modified from Ansah et al.

**Table 2:** Values of latency time, sedation time and recovery time in 18 felines sedated with the association of dexmedetomidine (7.5 ug/kg) and methadone (0.3 mg/kg) (DMT, *n* = 9) or dexmedetomidine (5 ug/kg), methadone (0.3 mg/kg), and midazolam (0.3 mg/kg), both intramuscular.

	DMT	DMTM
Latency time (minutes)	7.29 ± 3.18	7.55 ± 1.02
Sedation time (minutes)	29.66 ± 8.98	36.78 ± 9.43
Recovery time (minutes)	2.55 ± 4.42 A	5.55 ± 3.97 B

Values are presented as mean ± standard deviation. A, B : different capital letters in the same line represent significant differences between groups (*p* < 0.05).

## Data Availability

Access to data is restricted. The link for the statement can be found at “https://journals.sagepub.com/doi/10.1177/1098612X17720327?url_ver=Z39.88-2003∼∼∼∼∼∼∼∼∼^∼^∼^∼^∼∼∼∼∼∼∼∼∼∼∼amp;rfr_id=ori%3Arid%3Acrossref.org∼∼∼∼∼∼∼∼∼^∼^∼^∼^∼∼∼∼∼∼∼∼∼∼∼amp;rfr_dat=cr_pub++0pubmed∼∼∼∼∼∼∼∼∼^∼^∼^∼^∼∼∼∼∼∼∼∼∼∼∼amp;” and “https://avmajournals.avma.org/view/journals/javma/222/1/javma.2003.222.37.xml”
